# Baseline fibroblast growth factor 23 is associated with long-term mortality in ST-elevation myocardial infarction—results from the augsburg myocardial infarction registry

**DOI:** 10.3389/fcvm.2023.1173281

**Published:** 2023-08-04

**Authors:** Timo Schmitz, Bastian Wein, Margit Heier, Annette Peters, Christa Meisinger, Jakob Linseisen

**Affiliations:** ^1^ Epidemiology, Medical Faculty, University of Augsburg, Augsburg, Germany; ^2^ Department of Cardiology, Respiratory Medicine and Intensive Care, University Hospital Augsburg, Augsburg, Germany; ^3^ KORA Study Centre, University Hospital of Augsburg, Augsburg, Germany; ^4^ Helmholtz Zentrum München, Institute for Epidemiology, Neuherberg, Germany; ^5^ Chair of Epidemiology, Institute for Medical Information Processing, Biometry and Epidemiology, Medical Faculty, Ludwig-Maximilians-Universität München, Munich, Germany; ^6^ German Center for Diabetes Research (DZD), Neuherberg, Germany; ^7^ German Research Center for Cardiovascular Research (DZHK), Partner Site Munich Heart Alliance, Munich, Germany

**Keywords:** FGF-23, inflammatory plasma protein, myocardial infarction, STEMI, long-term mortality

## Abstract

**Background:**

The aim of this study was to investigate the association between inflammatory plasma protein concentrations and long-term mortality in patients with ST-elevation myocardial infarction (STEMI).

**Methods:**

For 343 STEMI patients recorded between 2009 and 2013 by the population-based Myocardial Infarction Registry Augsburg, 92 inflammatory plasma proteins were measured at the index event using the OLINK inflammation panel. In multivariable-adjusted Cox regression models, the association between each plasma protein and all-cause long-term mortality was investigated. Median follow-up time was 7.6 (IQR: 2.4) years. For plasma protein that showed a strong association with long-term mortality, a 5-year survival ROC analysis was performed.

**Results:**

One plasma protein, namely Fibroblast Growth Factor 23 (FGF-23), was particularly well associated with long-term mortality in the multivariable-adjusted Cox model with an FDR-adjusted *p*-value of <0.001 and a Hazard Ratio (HR) of 1.57 [95% CI: 1.29–1.91]. In the 5-years ROC analysis, an AUC of 0.6903 [95% CI: 0.594–0.781] was estimated for FGF-23. All other plasma protein didńt show strong associations, each marker with FDR-adjusted *p*-values >0.05 in the multivariable-adjusted Cox models.

**Conclusions:**

FGF-23 is independently associated with long-term mortality after STEMI and might play an important role in the response to myocardial injury. The results suggest FGF-23 to be a useful marker in the long-term treatment of STEMI patients and a potential target for drug development.

## 1. Background

Several prior studies investigated the association between a variety of inflammatory biomarkers and mortality after acute myocardial infarction (AMI) (1–6). Nevertheless, most studies concentrated on short-term morality or short-term major adverse cardiac events (MACE). There is a lack of evidence on whether inflammatory plasma proteins included in proteomics approaches, are associated with long-term outcomes in AMI patients. Thus, the aim of this study was to examine the association between a range of inflammation-related plasma proteins and long-term all-cause mortality in ST-elevation myocardial infarction (STEMI) patients and to identify the plasma proteins with the highest predictive ability for long-term mortality after the acute event.

## 2. Methods

### 2.1. Study population

This study is based on data from the population-based Augsburg Myocardial Infarction Registry. It was established in 1984 as a part of the MONICA-project (Monitoring Trends and Determinants in Cardiovascular disease) and operated as KORA Myocardial Infarction Registry (7) until 2019. The study area consists of the city of Augsburg, Germany, and the two adjacent counties comprising a total of approximately 680,000 inhabitants. Patients aged between 25 and 84 years being admitted for an AMI to one out of eight hospitals in the study area were consecutively registered. More detailed information on case identification, diagnostic classification of events and quality control of the data can be found in previous publications (7, 8). For the present study, only patients with AMI admitted to the university hospital of Augsburg between 2009 and 2014 were included, since blood from AMI patients was collected solely in this hospital. All study participants gave written informed consent. Methods of data collection have been approved by the ethics committee of the Bavarian Medical Association (Bayerische Landesaerztekammer) and the study was performed in accordance with the Declaration of Helsinki. The Augsburg Myocardial Infarction Registry is registered at “Deutsches Register Klinischer Studien” (DRKS) with the project number DRKS00029042.

#### 2.1.1. Data collection

Trained study nurses interviewed the participants during hospital stay using a standardized questionnaire. In order to confirm the information provided by the patients and to collect additional information, the patients' medical chart was reviewed. Demographic data, data on cardiovascular risk factors, medical history, comorbidities (including diabetes) and medication before and during hospital stay, as well as at discharge were collected from each patient. Furthermore, routine laboratory parameters including glucose measurement, ECG results and data on the in-hospital course were assessed.

Between 2009 and 2014 arterial EDTA blood samples were obtained immediately after hospital admission, right at the beginning of the cardiac catheterization. After centrifugation and aliqoting in the catheter laboratory, EDTA plasma samples were stored frozen at −80°C until laboratory analysis.

#### 2.1.2. Clinical chemistry measurement

Measurements of the 92 proteins were performed in plasma samples using the Proseek® Multiplex Inflammation panel, developed by Olink Proteomics (Uppsala, Sweden) and based on the Proximity Extension Assay (PEA) technology. Detailed information on the process of measurement can be found in a previous publication (9) and directly at the website of Olink Proteomics (10).

All other blood parameters were measured in clinical laboratory at the university hospital of Augsburg during hospital stay of the patients as part of the regular diagnosis and routine treatment.

#### 2.1.3. Outcome

The endpoint used in this study was long-term all-cause mortality. Mortality was ascertained by regularly checking the vital status of all registered persons of the MI registry with data from the population registries. Death certificates were obtained from local health departments. The present analysis included patients with ST elevation myocardial infarction (STEMI) only. All patients who died within the first 28 days after AMI were excluded in order to concentrate on long-term mortality exclusively. Overall, 343 STEMI patients survived the first 28 days after infarction and were considered for this analysis.

### 2.2. Statistical analysis

For the comparison of categorical variables, *χ*^2^ tests were performed and the results were presented as absolute frequencies with percentages. For normally-distributed continuous variables, Student's *t*-tests were used. For continuous variables that were not normally-distributed we used nonparametric tests. The results were presented as mean and standard deviation (SD) or median and inter-quartiles range (IQR).

#### 2.2.1. Cox regression models

First, the obtained values for each plasma protein were standardized in order to ensure comparability between the different markers. To examine the associations between the 92 plasma proteins and long-term mortality, 92 Cox regression models were calculated, one for each protein. The initial models were adjusted for sex and age. To control the effect of multiple testing, the obtained *p*-values were false discovery rate (FDR)-adjusted. In a subsequent step, the same Cox regression models were calculated but potential confounder variables were added according to a literature review. These multivariable-adjusted Cox regression models were adjusted for sex, age, glomerular filtration rate (GFR; continuous variable), diabetes mellitus, hypertension, hyperlipidemia and percutaneous coronary intervention (PCI), yes/no).

The proportional hazards assumption was checked by plotting the Schoenfeld residuals against time and searching for any visible correlation and by testing for a significant correlation of the Schoenfeld residuals with time. The proportional hazards assumption was considered to be sufficiently fulfilled for all models.

As a sensitivity analysis, two more multivariable adjusted Cox regression models were calculated, each including the same covariables as the main model but adding either prehospital time or left ventricular ejection fraction (LVEF) (see Supplementary Figures S1, S2). Finally, in these models, it was tested for a significant interaction between the plasma proteins and prehospital or LVEF time, respectively, by including an interaction term into the Cox models.

#### 2.2.2. ROC analysis, Youden-Index, and Kaplan-Meier curve

For plasma proteins that were strongly associated with long-term mortality ROC analyses was performed to quantify their predictive ability.

Best cut-off value for a dichotomization of the plasma protein markers were determined by calculating Youden-Indices. The sample then was divided in a “high plasma protein” group and a “low plasma protein” group according to the maximized Youden-Index. Finally, a Kaplan-Meier curve was plotted and stratified by the dichotomous variable high/low plasma protein group.

## 3. Results

For a total of 343 STEMI cases data of 92 inflammation-related plasma proteins were available for the statistical analysis. Table 1 displays the baseline characteristics of the total sample and stratified for patients who died within the observational period and those who didn’t.

**Table 1:** Baseline characteristics for the total sample and stratified for event/no event (event = death 28 days after AMI and within observational period).

	Total sample (*n* = 343)	No event (*n* = 257)	Event (*n* = 86)	*p*-value	*n*
Age (mean, SD)	62.8 (11.8)	60.1 (11.1)	70.8 (10.1)	<0.001	343
Male	252 (73.5)	192 (74.7)	60 (69.8)	0.4489	343
Comorbidities					
Hypertension	259 (75.5)	183 (71.2)	76 (88.4)	0.0022	343
Diabetes mellitus	92 (26.8)	57 (22.2)	35 (40.7)	0.0013	343
Hyperlipidemia	198 (57.7)	149 (58)	49 (57)	0.9710	343
Smoking status				0.0265	338
Current smoker	135 (39.9)	113 (44)	22 (27.2)	–	–
Ex-smoker	99 (29.3)	70 (27.2)	29 (35.8)	–	–
Never smoker	104 (30.8)	74 (28.8)	30 (37)	–	–
Clinical characteristics					
Prehospital time in minutes (median, IQR)	120 (70–273.5)	125 (80–271)	103 (58.5–277.5)	0.0554	324
Systolic blood pressure at admission (median, IQR)	144 (128–160)	140 (125–160)	148 (130.5–163.25)	0.1722	343
Diastolic blood pressure at admission (median, IQR)	80 (70–97)	80 (70–100)	80 (70–94)	0.1569	340
Heart rate at admission (median, IQR)	76 (67–88)	76 (66.75–88)	80 (67–90)	0.1517	341
Left ventricular EF				0.0042	333
>50%	163 (48.9)	131 (52.6)	32 (38.1)	–	–
41–50%	75 (22.5)	58 (23.3)	17 (20.2)	–	–
31–40%	74 (22.2)	50 (20.1)	24 (28.6)	–	–
≤30%	21 (6.3)	10 (4)	11 (13.1)	–	–
Kidney function				<0.001	343
eGFR ≥60 ml/min/1.73 m^2^	247 (72)	202 (78.6)	45 (52.3)	–	–
eGFR 30–59 ml/min/1.73 m^2^	85 (24.8)	54 (21)	31 (36)	–	–
eGFR < 30 ml/min/1.73 m^2^	11 (3.2)	1 (0.4)	10 (11.6)	–	–
Treatment					
PCI	314 (91.5)	240 (93.4)	74 (86)	0.0583	343
Bypass therapy	34 (9.9)	22 (8.6)	12 (14)	0.2149	343
Lysis therapy	2 (0.6)	2 (0.8)	0 (0)	0.9981	343
Laboratory values					
Troponin I at admission (median, IQR)	0.555 (0.08–4.9525)	0.490 (0.0775–4.505)	1.085 (0.0975–6.6525)	0.1388	336
peak CRP (median, IQR)	0.37 (0.29–0.985)	0.34 (0.29–0.81)	0.54 (0.29–1.55)	0.0081	342

**Figure 1:**
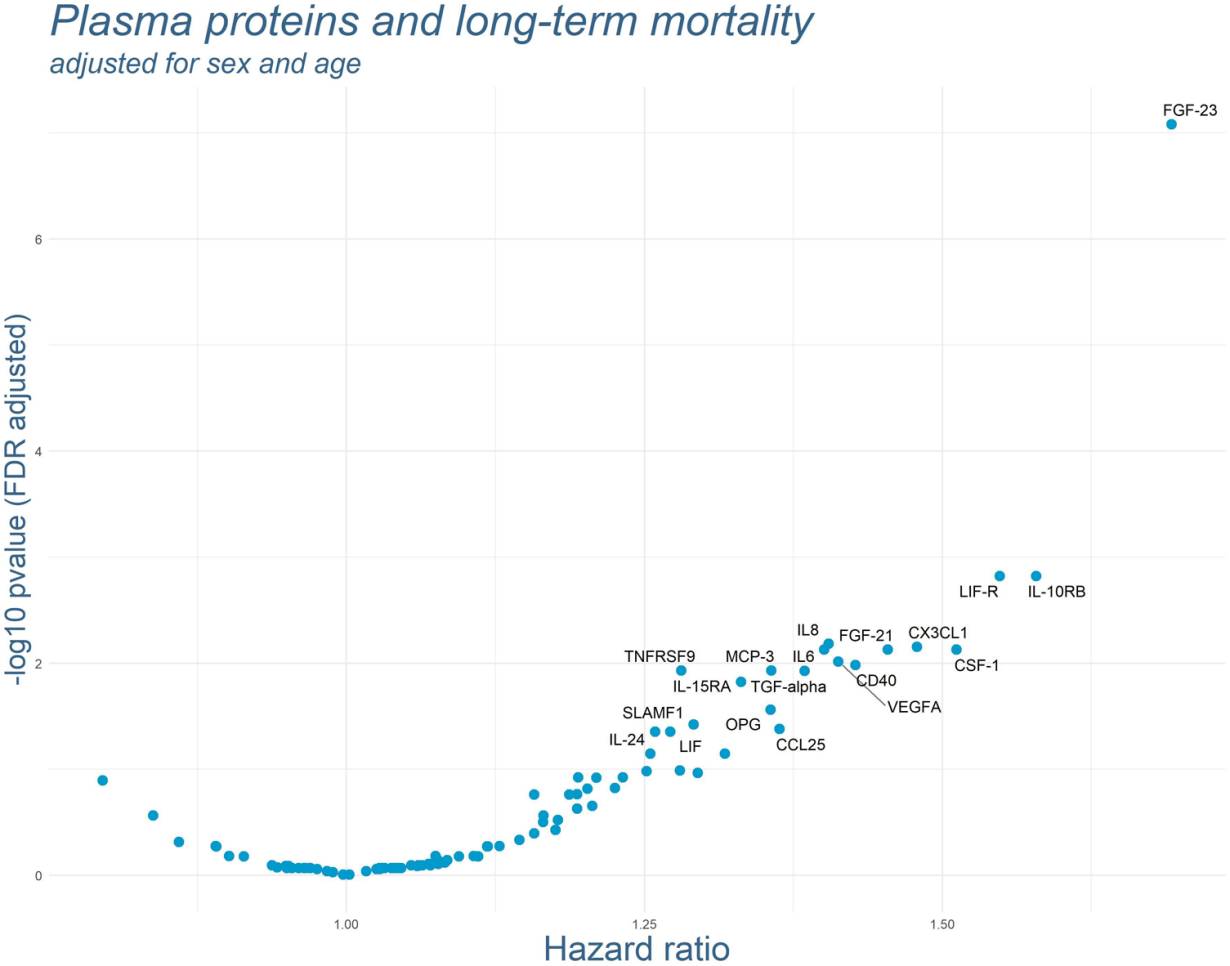
Results of the Cox regression models for each plasma protein adjusted for sex and age. *P*-values were FDR-adjusted. Names of the markers are presented for all markers with FDR-adjusted *p*-values below 0.05. Cases with death within the first 28 day after AMI were excluded.

**Figure 2:**
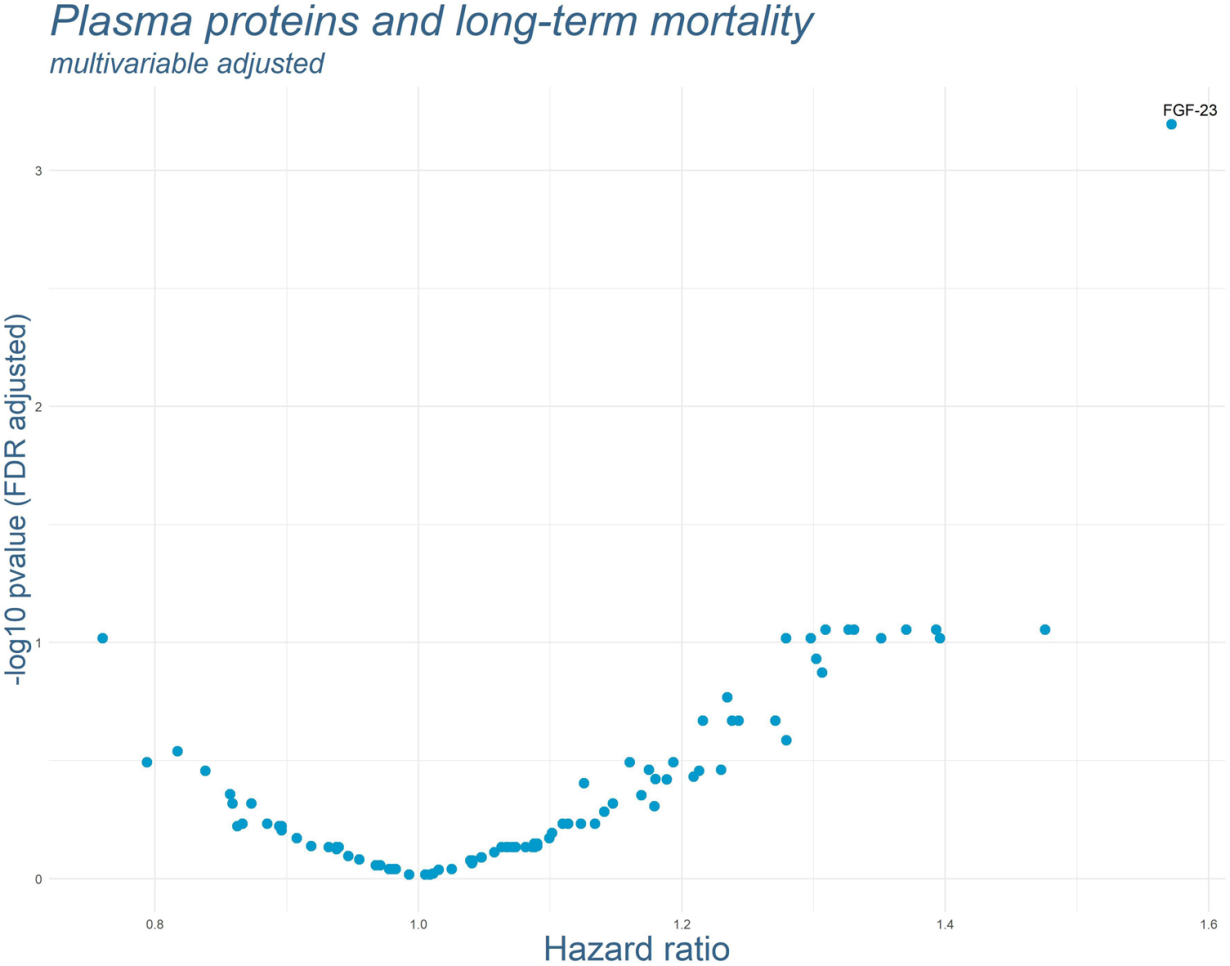
Results of the multivariable adjusted Cox regression models for each plasma protein. *P*-values were FDR-adjusted. Names of the proteins are presented for all markers with FDR-adjusted *p*-values below 0.05. (Adjusted for: sex, age, renal function according to GFR, diabetes mellitus, hypertension, hyperlipidemia and PCI).

As for most plasma proteins 3 values were missing, a total of 340 cases was included into the Cox regression models (there were no missing values for the covariables included into the main regression models). Results of the Cox regression models adjusted for sex and age are displayed in Figure 1. One protein, Fibroblast Growth Factor 23 (FGF-23), was strikingly associated with long-term mortality. Adjusting the Cox regression model for sex, age, GFR, diabetes mellitus, hypertension, hyperlipidemia and PCI did not change the association substantially (see Figure 2). In this model, FGF-23 produced an FDR-adjusted *p*-value of <0.001 and a Hazard Ratio (HR) of 1.57 [95% CI: 1.29–1.91] per SD.

**Figure 3:**
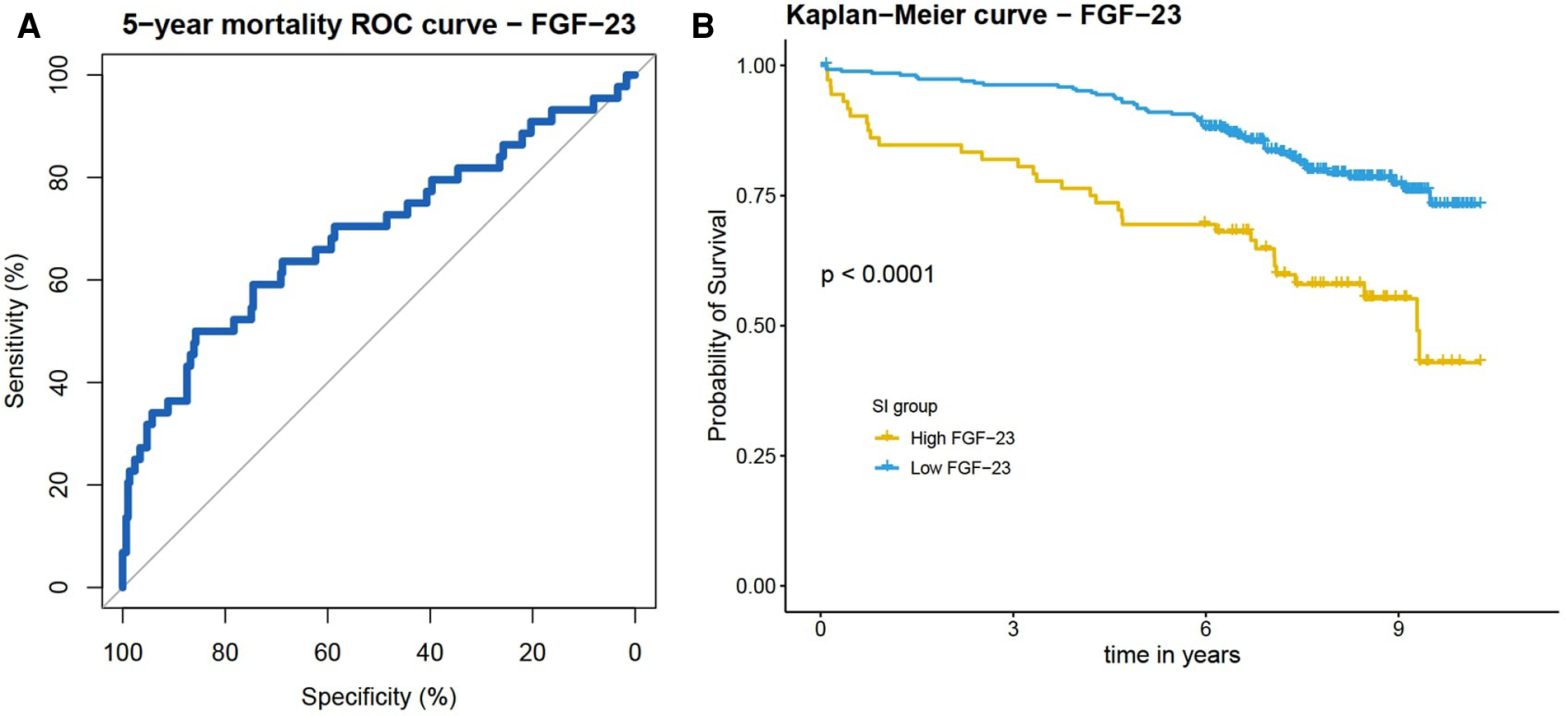
(**A**) ROC curve for 5-years mortality. Plasma FGF-23 showed good predictive ability with an AUC of 0.6903. (**B**) Kaplan-Meier plot stratified for high and low FGF-23 groups (defined by Youden-Index and a cut-off value of 0.4186204 SD).

In the subsequently performed 5-years ROC analysis, 339 patients were included (for 1 patient there was no information on 5 year survival). The number of events (=death within 5 years) was 44 and the number of controls was 295. Figure 3A shows the ROC curve for FGF-23, and the AUC was 0.6903 [95% CI: 0.5937–0.781]. On the basis of the ROC analysis we calculated the optimal cut-off value for a dichotomization of the standardized FGF-23 variable, which was at 0.4186204 SD (Youden-Index: 1.357627). Using this cut-off for categorization of the full study sample, 64 patients were assigned to the “high FGF-23 group” and 276 patients were assigned to the “low FGF-23 group”. Figure 3B displays the Kaplan-Meier curves of those two groups (high/low FGF-23 group).

## 4. Discussion

In this study, we investigated the association of 92 plasma proteins with long-term mortality in ST-elevation myocardial infarction patients. One protein, FGF-23, exhibited a particularly strong association with long-term mortality in the multivariable-adjusted Cox regression model (HR: 1.57, 95% CI: 1.29–1.91) as well as in a 5 years ROC analysis (AUC: 0.6903, 95% CI: 0.5937–0.781). All other plasma proteins were not significantly related to long-term mortality in the multivariable-adjusted Cox regression model.

FGF-23 is a representative of the fibroblast growth factor family and is classified by OLINK as a cancer marker. It is mainly produced by osteoblasts and osteocytes but can also be produced and secreted by other tissue cells (11). Several prior studies reported associations between FGF-23 and mortality in the general population (12–15). There is also strong evidence for an association between elevated FGF-23 concentrations and cardiovascular events in general and mortality in coronary artery disease (CAD) and after AMI in particular (16–20). Nevertheless, the majority of studies on FGF-23 and mortality in CAD patients and after AMI used short- to mid-term mortality as the primary endpoint and so it remains unclear, whether FGF-23 concentrations are also associated with long-term mortality after AMI and whether the effects remain stable over the course of several years. With a median follow-up time of 7.6 years, this study was able to address this question indicating a long-lasting association up to one decade after the acute event.

### 4.1. Causality of the relationship

In a meta-analysis on FGF-23 and risks of cardiovascular and non-cardiovascular diseases the authors concluded that the association between FGF-23 and the diseases might be non-causal (21). In a rat model, Andrukhova et al. found that the induction of myocardial infarction in rats led to an elevation of FGF-23 concentrations (22), which strongly indicates a principal relation between AMI and FGF-23. Likewise, Schumacher et al. reported that in mice cardiac fibroblasts located in infarcted myocardial tissue produced and expressed FGF-23 in response to the induced AMI (23).Thus, results of these studies suggest that AMI causes an elevation of plasma FGF-23 concentrations and do not support the idea of elevated FGF-23 concentrations being a risk factor for AMI and AMI-related mortality. In a further study, Hao et al. investigated the role of FGF-23 in post-infarct myocardial fibrosis in mice. Their findings indicate that FGF-23 actually promotes myocardial fibrosis and exacerbates diastolic dysfunction induced by MI. To sum up, the scientific data on the causal nature of the relationship between FGF-23 and AMI is not very clear at the moment.

### 4.2. FGF-23 and left ventricular (LV) remodeling

It has been reported that high plasma FGF-23 concentrations are independently associated with LV remodeling after STEMI (24). Pathological ventricular remodeling is a major complication after AMI and affects cardiac function and overall prognosis in the long-term (25). Likely, pathological ventricular remodeling plays a major role in the association between elevated FGF-23 plasma concentrations and long-term mortality in AMI patients. Nevertheless, in our study no data on ventricular remodeling over the course of the years was available and therefore we are not able to further examine these supposed interconnections.

### 4.3. FGF-23 and chronic kidney disease (CKD)

Other pathophysiological pathways might potentially be involved in the association between plasma FGF-23 and long-term outcome after STEMI. First, FGF-23 is known to be strongly connected to CKD, which is a major risk factor for adverse outcomes in AMI patients (26–29). Consequently, it could be speculated that the predictive ability of FGF-23 for long-term outcome in STEMI patients might substantially be driven by the association between FGF-23 and kidney function. Nevertheless, the multivariable Cox regression model was adjusted for the numerical variable GFR (ml/min/1.73 m^2^), so we conclude that the association between FGF-23 and mortality after AMI is (mainly) independent of renal function or CKD.

### 4.4. Heart failure and reduced left-ventricular ejection fraction (LVEF)

The scientific literature moreover suggests a relation between FGF-23 and heart failure/reduced left-ventricular ejection fraction (30–32). FGF-23 is also a strong predictor of outcome in heart failure patients (33–35). It is well known, that impaired LVEF goes along with higher long-term mortality in AMI patients (36, 37). In a study by Cornelissen et al., FGF-23 was only predictive for 1-year mortality in AMI patients with heart failure, but not so in AMI patients without heart failure; which indicates an interaction with heart failure and LVEF. Thus, we tested this hypothesis but couldn’t find a significant interaction between LVEF and FGF-23 plasma concentrations in our data. We moreover hypothesized, that the association between elevated FGF-23 levels and long-term mortality after AMI could partially be mediated by a reduced LVEF. So we calculated a multivariable Cox regression model including LVEF (see Supplementary Figure S1), but again, there was almost no change to the original model.

### 4.5. Prehospital time and therapeutic delay

The blood samples in this study were very uniformly taken at the beginning of the cardiac catheterization. As we included only STEMI events, the delay between hospital admission and PCI was generally very small. Nevertheless, greater deviations between the patients were observed in prehospital time, which represent the majority of therapeutic delay and negatively affects outcome in AMI patients (38, 39). Takahshi et al. found that serum FGF-23 concentrations after AMI treated with PCI follow a certain course of time after AMI with a slight decrease on day 1 and 3 after admission and an increase on day 5 and 7 (40); Thorsen et al. came to similar results reporting a reduction in FGF-23 concentrations during the acute phase of AMI and a normalization after seven days following successful revascularization (41). To take a closer look at this and in order to examine whether the association between FGF-23 and mortality was mainly driven by the therapeutic delay, we calculated another multivariable adjusted model including prehospital time in minutes (see Supplementary Figure S2). Although results of the model were influenced to a limited extent, it shows that therapeutic delay does not play a superior role in the association between FGF-23 and long-term mortality.

### 4.6. Strengths and limitation

This study is characterized by some strengths. First, it is based on patients from the population-based Augsburg Myocardial Infarction Registry with consecutive enrollment of patients, which minimizes the effect of selection bias. Blood samples were uniformly taken during the PTCA intervention, guaranteeing highest consistency in blood sampling. Moreover, for every case there was a large number of additional information which we used for proper adjustment in the Cox regression models.

Nevertheless, there are also some limitations. We have no validation cohort from another register for our analyses, which means we cannot validate the associations found in this study. Moreover, as this study is based solely on observational data, we cannot draw any conclusions about causality (including the possibility of reverse causality). Additionally, we might not have considered all potential confounders. Last, as this study included only patients between 25 and 84 years with STEMI, the results may not be generalized to all age or ethnic groups as well as to Non-ST-elevation MI events.

## 5. Conclusion

Elevated FGF-23 plasma concentrations are independently associated with higher long-term mortality in STEMI patients. The effects appear to remain stable over the course of one decade. FGF-23-concentrations at baseline might help to identify a population in the need of intensive and lasting secondary prevention measures to target long-term mortality. Moreover, if a causal nature of the relationship can be established, new therapies targeting FGF-23 might represent a promising approach especially in the long-term treatment of patients after AMI.

## Data Availability

The datasets generated during and/or analyzed in the current study are not publicly available due to data protection aspects but are available in an anonymized form from the corresponding author on reasonable request. Requests to access these datasets should be directed to TS, timo.schmitz@med.uni-augsburg.de.

## References

[1] Scirica BM Sabatine MS Jarolim P Murphy SA Lemos Jd Braunwald E Assessment of multiple cardiac biomarkers in non-ST-segment elevation acute coronary syndromes: observations from the MERLIN-TIMI 36 trial. Eur Heart J. (2011) 32:697–705. 10.1093/eurheartj/ehq468 21183500 PMC6279197

[2] O'Malley RG Bonaca MP Scirica BM Murphy SA Jarolim P Sabatine MS Prognostic performance of multiple biomarkers in patients with non-ST-segment elevation acute coronary syndrome: analysis from the MERLIN-TIMI 36 trial (metabolic efficiency with ranolazine for less ischemia in non-ST-elevation acute coronary syndromes-thrombolysis in myocardial infarction 36). J Am Coll Cardiol. (2014) 63:1644–53. 10.1016/j.jacc.2013.12.034 24530676

[3] Oemrawsingh RM Lenderink T Akkerhuis KM Heeschen C Baldus S Fichtlscherer S Multimarker risk model containing troponin-T, interleukin 10, myeloperoxidase and placental growth factor predicts long-term cardiovascular risk after non-ST-segment elevation acute coronary syndrome. Heart. (2011) 97:1061–6. 10.1136/hrt.2010.197392 21558475

[4] O'Donoghue ML Morrow DA Cannon CP Jarolim P Desai NR Sherwood MW Multimarker risk stratification in patients with acute myocardial infarction. J Am Heart Assoc. (2016) 5:1–9. 10.1161/JAHA.115.002586 PMC4889163 27207959

[5] Hjort M Eggers KM Lindhagen L Baron T Erlinge D Jernberg T Differences in biomarker concentrations and predictions of long-term outcome in patients with ST-elevation and non-ST-elevation myocardial infarction. Clin Biochem. (2021) 98:17–23. 10.1016/j.clinbiochem.2021.09.001 34496288

[6] Skau E Henriksen E Wagner P Hedberg P Siegbahn A Leppert J. GDF-15 and TRAIL-R2 are powerful predictors of long-term mortality in patients with acute myocardial infarction. Eur J Prev Cardiol. (2017) 24:1576–83. 10.1177/2047487317725017 28762762

[7] Löwel H Meisinger C Heier M Hörmann A. The population-based acute myocardial infarction (AMI) registry of the MONICA/KORA study region of augsburg. Gesundheitswesen. (2005) 67(Suppl 1):S31–7. 10.1055/s-2005-858241 16032515

[8] Kuch B Heier M Scheidt Wv Kling B Hoermann A Meisinger C. 20-year Trends in clinical characteristics, therapy and short-term prognosis in acute myocardial infarction according to presenting electrocardiogram: the MONICA/KORA AMI registry (1985–2004). J Intern Med. (2008) 264:254–64. 10.1111/j.1365-2796.2008.01956.x 18397247

[9] Ponce-de-Leon M Linseisen J Peters A Linkohr B Heier M Grallert H Novel associations between inflammation-related proteins and adiposity: a targeted proteomics approach across four population-based studies. Transl Res. (2022) 242:93–104. 10.1016/j.trsl.2021.11.004 34780968

[10] Olink Proteomics. *Normalized Protein eXpression*. Available at: https://www.olink.com/faq/what-is-npx/

[11] Hu MC Shiizaki K Kuro-o M Moe OW. Fibroblast growth factor 23 and Klotho: physiology and pathophysiology of an endocrine network of mineral metabolism. Annu Rev Physiol. (2013) 75:503–33. 10.1146/annurev-physiol-030212-183727 23398153 PMC3770142

[12] Souma N Isakova T Lipiszko D Sacco RL Elkind MS DeRosa JT Fibroblast growth factor 23 and cause-specific mortality in the general population: the northern manhattan study. J Clin Endocrinol Metab. (2016) 101:3779–86. 10.1210/jc.2016-2215 27501282 PMC5052338

[13] Lutsey PL Alonso A Selvin E Pankow JS Michos ED Agarwal SK Fibroblast growth factor-23 and incident coronary heart disease, heart failure, and cardiovascular mortality: the atherosclerosis risk in communities study. J Am Heart Assoc. (2014) 3:e000936. 10.1161/JAHA.114.000936 24922628 PMC4309096

[14] Ärnlöv J Carlsson AC Sundström J Ingelsson E Larsson A Lind L Higher fibroblast growth factor-23 increases the risk of all-cause and cardiovascular mortality in the community. Kidney Int. (2013) 83:160–6. 10.1038/ki.2012.327 22951890

[15] Ix JH Katz R Kestenbaum BR Boer Id Chonchol M Mukamal KJ Fibroblast growth factor-23 and death, heart failure, and cardiovascular events in community-living individuals: CHS (cardiovascular health study). J Am Coll Cardiol. (2012) 60:200–7. 10.1016/j.jacc.2012.03.040 22703926 PMC3396791

[16] Batra J Buttar RS Kaur P Kreimerman J Melamed ML. FGF-23 and cardiovascular disease: review of literature. Curr Opin Endocrinol Diabetes Obes. (2016) 23:423–9. 10.1097/MED.0000000000000294 27652999 PMC6936216

[17] Parker BD Schurgers LJ Brandenburg VM Christenson RH Vermeer C Ketteler M The associations of fibroblast growth factor 23 and uncarboxylated matrix Gla protein with mortality in coronary artery disease: the heart and soul study. Ann Intern Med. (2010) 152:640–8. 10.7326/0003-4819-152-10-201005180-00004 20479029 PMC3079370

[18] Schumacher D Schuh A. Cardiac FGF23: a new player in myocardial infarction. Discoveries (Craiova). (2019) 7:e97. 10.15190/d.2019.10 32309615 PMC7086082

[19] Eggers KM Lindhagen L Baron T Erlinge D Hjort M Jernberg T Sex-differences in circulating biomarkers during acute myocardial infarction: an analysis from the SWEDEHEART registry. PLoS One. (2021) 16:e0249830. 10.1371/journal.pone.0249830 33831096 PMC8031406

[20] Vázquez-Sánchez S Poveda J Navarro-García JA González-Lafuente L Rodríguez-Sánchez E Ruilope LM An overview of FGF-23 as a novel candidate biomarker of cardiovascular risk. Front Physiol. (2021) 12:632260. 10.3389/fphys.2021.632260 33767635 PMC7985069

[21] Marthi A Donovan K Haynes R Wheeler DC Baigent C Rooney CM Fibroblast growth factor-23 and risks of cardiovascular and noncardiovascular diseases: a meta-analysis. J Am Soc Nephrol. (2018) 29:2015–27. 10.1681/ASN.2017121334 29764921 PMC6050929

[22] Andrukhova O Slavic S Odörfer KI Erben RG. Experimental myocardial infarction upregulates circulating fibroblast growth factor-23. J Bone Miner Res. (2015) 30:1831–9. 10.1002/jbmr.2527 25858796 PMC4973700

[23] Schumacher D Alampour-Rajabi S Ponomariov V Curaj A Wu Z Staudt M Cardiac FGF23: new insights into the role and function of FGF23 after acute myocardial infarction. Cardiovasc Pathol. (2019) 40:47–54. 10.1016/j.carpath.2019.02.001 30852297

[24] Reindl M Reinstadler SJ Feistritzer H-J Mueller L Koch C Mayr A Fibroblast growth factor 23 as novel biomarker for early risk stratification after ST-elevation myocardial infarction. Heart. (2017) 103:856–62. 10.1136/heartjnl-2016-310520 27979879

[25] Bhatt AS Ambrosy AP Velazquez EJ. Adverse remodeling and reverse remodeling after myocardial infarction. Curr Cardiol Rep. (2017) 19:71. 10.1007/s11886-017-0876-4 28660552

[26] Komaba H Fukagawa M. The role of FGF23 in CKD–with or without Klotho. Nat Rev Nephrol. (2012) 8:484–90. 10.1038/nrneph.2012.116 22714041

[27] Scialla JJ Xie H Rahman M Anderson AH Isakova T Ojo A Fibroblast growth factor-23 and cardiovascular events in CKD. J Am Soc Nephrol. (2014) 25:349–60. 10.1681/ASN.2013050465 24158986 PMC3904568

[28] Di Giuseppe R Kühn T Hirche F Buijsse B Dierkes J Fritsche A Plasma fibroblast growth factor 23 and risk of cardiovascular disease: results from the EPIC-Germany case-cohort study. Eur J Epidemiol. (2015) 30:131–41. 10.1007/s10654-014-9982-4 25527370

[29] Seiler S Reichart B Roth D Seibert E Fliser D Heine GH. FGF-23 and future cardiovascular events in patients with chronic kidney disease before initiation of dialysis treatment. Nephrol Dial Transplant. (2010) 25:3983–9. 10.1093/ndt/gfq309 20525642

[30] Maaten Jt Voors AA Damman K van der Meer P Anker SD Cleland JG Fibroblast growth factor 23 is related to profiles indicating volume overload, poor therapy optimization and prognosis in patients with new-onset and worsening heart failure. Int J Cardiol. (2018) 253:84–90. 10.1016/j.ijcard.2017.10.010 29306478

[31] Jeinsen BV Sopova K Palapies L Leistner DM Fichtlscherer S Seeger FH Bone marrow and plasma FGF-23 in heart failure patients: novel insights into the heart-bone axis. ESC Heart Fail. (2019) 6:536–44. 10.1002/ehf2.12416 30912310 PMC6487718

[32] Plischke M Neuhold S Adlbrecht C Bielesz B Shayganfar S Bieglmayer C Inorganic phosphate and FGF-23 predict outcome in stable systolic heart failure. Eur J Clin Invest. (2012) 42:649–56. 10.1111/j.1365-2362.2011.02631.x 22150123

[33] Duran A daSilva-deAbreu A Joury A Ventura HO. FGF23 Predicts outcomes in heart failure but questions remain unanswered. Int J Cardiol. (2021) 338:145–6. 10.1016/j.ijcard.2021.06.036 34157358

[34] Vergaro G Aimo A Taurino E Del Franco A Fabiani I Prontera C Discharge FGF23 level predicts one year outcome in patients admitted with acute heart failure. Int J Cardiol. (2021) 336:98–104. 10.1016/j.ijcard.2021.05.028 34019969

[35] Gruson D Lepoutre T Ketelslegers J-M Cumps J Ahn SA Rousseau MF. C-terminal FGF23 is a strong predictor of survival in systolic heart failure. Peptides. (2012) 37:258–62. 10.1016/j.peptides.2012.08.003 22902597

[36] Bosch X Théroux P. Left ventricular ejection fraction to predict early mortality in patients with non-ST-segment elevation acute coronary syndromes. Am Heart J. (2005) 150:215–20. 10.1016/j.ahj.2004.09.027 16086920

[37] Syyli N Hautamäki M Antila K Mahdiani S Eskola M Lehtimäki T Left ventricular ejection fraction adds value over the GRACE score in prediction of 6-month mortality after ACS: the MADDEC study. Open Heart. (2019) 6:e001007. 10.1136/openhrt-2019-001007 31328004 PMC6609116

[38] Luca Gd van ‘t Hof AW Boer M-Jd Ottervanger JP Hoorntje JC Gosselink AT Time-to-treatment significantly affects the extent of ST-segment resolution and myocardial blush in patients with acute myocardial infarction treated by primary angioplasty. Eur Heart J. (2004) 25:1009–13. 10.1016/j.ehj.2004.03.021 15191770

[39] Wu J-R Moser DK Riegel B McKinley S Doering LV. Impact of prehospital delay in treatment seeking on in-hospital complications after acute myocardial infarction. J Cardiovasc Nurs. (2011) 26:184–93. 10.1097/JCN.0b013e3181efea66 21116191

[40] Takahashi H Ozeki M Fujisaka T Morita H Fujita S-I Takeda Y Changes in serum fibroblast growth factor 23 in patients with acute myocardial infarction. Circ J. (2018) 82:767–74. 10.1253/circj.CJ-17-0826 29151454

[41] Thorsen IS Gøransson LG Ueland T Aukrust P Manhenke CA Skadberg Ø The relationship between fibroblast growth factor 23 (FGF23) and cardiac MRI findings following primary PCI in patients with acute first time STEMI. Int J Cardiol Heart Vasc. (2021) 33:100727. 10.1016/j.ijcha.2021.100727 33665349 PMC7905449

