# Supplementary material for: Baseline fibroblast growth factor 23 is associated with long-term mortality in ST-elevation myocardial infarction—results from the augsburg myocardial infarction registry
Source: Front Cardiovasc Med. 2023 Aug 4;10:1173281. doi: 10.3389/fcvm.2023.1173281 (PMC10436601; doi:10.3389/fcvm.2023.1173281)
Supplement: Supplementary file 1 [file Datasheet1.pdf]

Baseline Fibroblast Growth Factor 23 is associated with long-term mortality in ST-elevation myocardial infarction – results from the Augsburg Myocardial Infarction Registry.

Schmitz T, Wein B, Heier M, Peters A, Meisinger C, Linseisen J

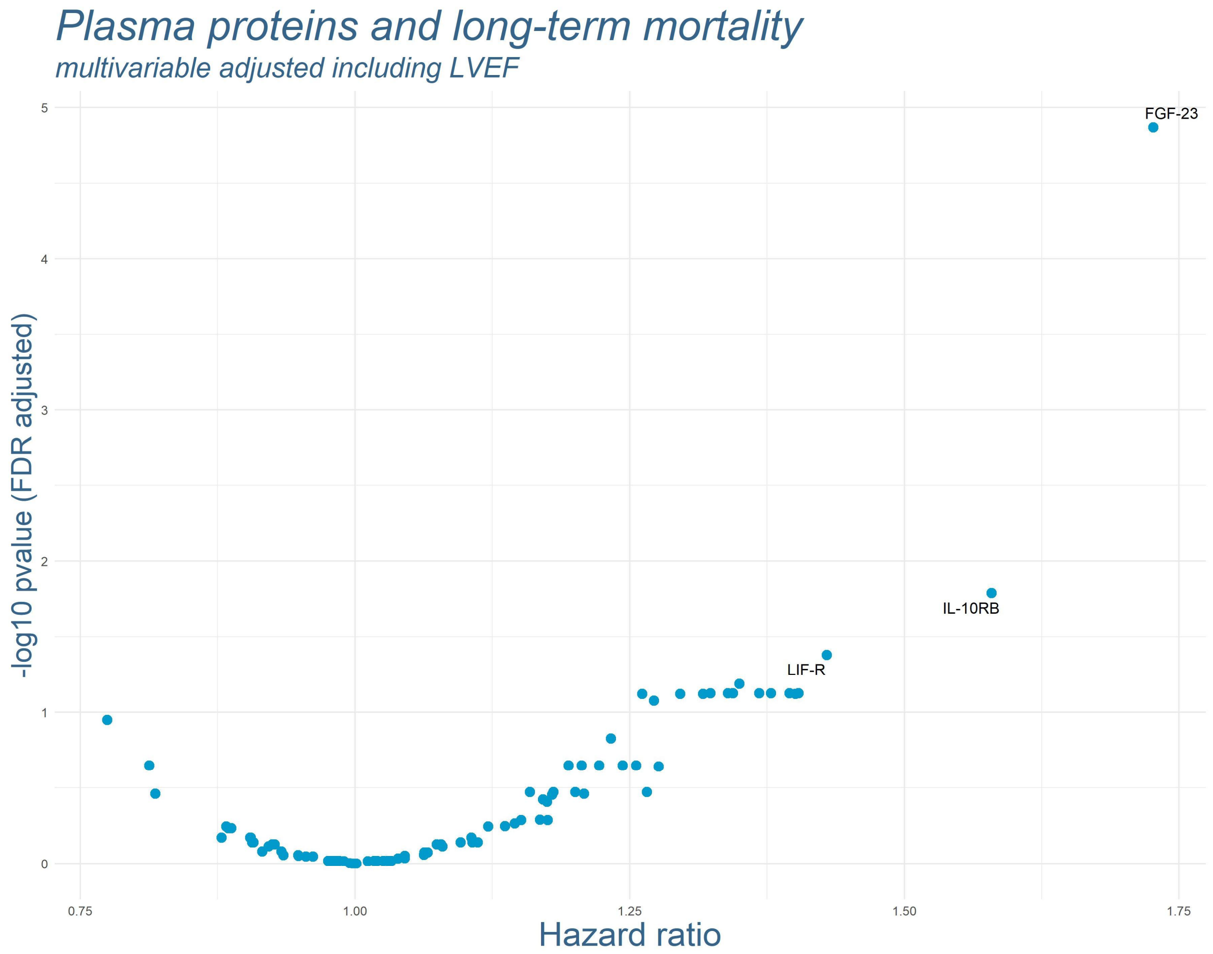

Figure S1: Results of the multivariable adjusted Cox regression models including prehospital time. P-values were FDR-adjusted. Names of the markers are presented for all markers with FDR-adjusted p-values below 0.05.

frontiers

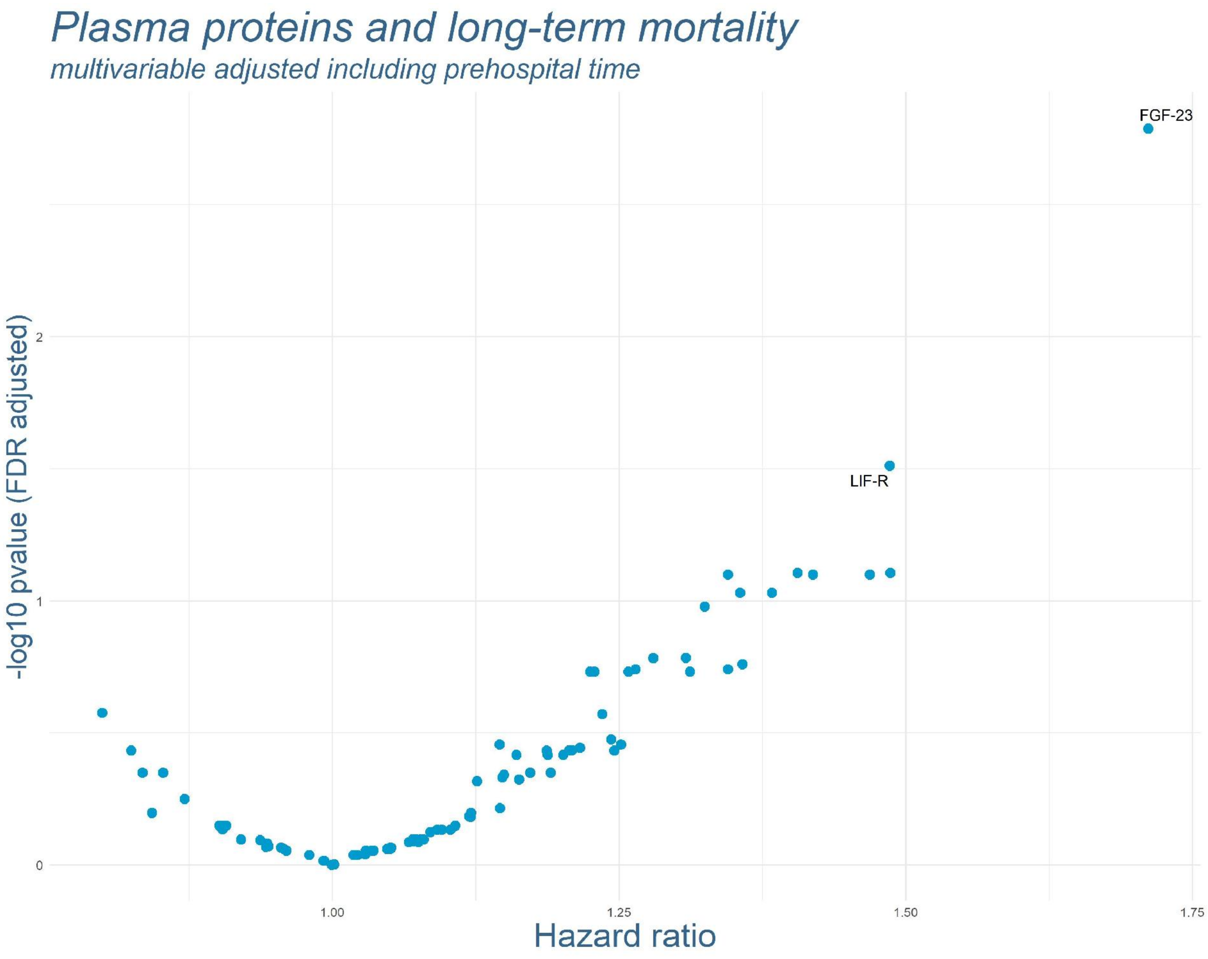

Figure S2: Results of the multivariable adjusted Cox regression models including prehospital time. P-values were FDR-adjusted. Names of the markers are presented for all markers with FDR-adjusted p-values below 0.05.
